# Annotations

**Published:** 1890-02-22

**Authors:** 


### Annotations.

**London Paupers to the Country.**

At first sight the proposal of the St. Pancras Guardians to remove their workhouse to the country seems altogether to command the approval of those who wish the aged and helpless poor to be well and happy. The pure air and wholesome sights and sounds of the country, as compared with the stuffy atmosphere and the noise and bustling excitement of the town, appear quite conclusive in favour of speedy removal. But Miss Julia Wedgwood, in a letter to the morning papers, puts quite a different aspect on the case. Miss Wedgwood points out that workhouse life is extremely dreary and monotonous. The visits of friends are to the aged and infirm poor occasions of such pleasure and excitement as the ordinary home-dweller can hardly understand. Those visits, which can be easily and frequently paid whilst the workhouse is in London and close at hand, must practically cease if each visit is to necessitate a considerable railway journey into the country. But Miss Wedgwood further points out that in London visitors and relatives can act the part of friendly critics and inspectors. The officials, without being too conscious of the fact, recognise those visits as constant and salutary checks. If, however, the workhouse were to be removed thirty or forty miles into the country, those checks would operate no longer, and officialism would have unlimited opportunities of practising its worst faults. We do not say that the St. Pancras officials have any faults at all; but we do affirm that all officials whatsoever are made better and kept better by the knowledge that their conduct is overlooked by persons who could be easily converted into unfriendly critics. Moreover, your born Londoner is always a Londoner, and in the large majority of cases prefers his beloved streets with their smoke and fog and noise to the finest landscape and the purest air that the country can produce. On the whole, therefore, the St. Pancras Guardians, even if they decide to remove their workhouse from its present site, will do well to consider if they cannot rebuild it in such a situation as shall preserve to the inmates the few but dearly-valued privileges which they now enjoy. It is bad enough in any case to be a pauper; it is much worse if in actual practice the pauper is also a prisoner.

**Benzon and his Friends.**

Physiologists and lunacy doctors take a growing interest in the various and peculiar manifestations of brain activity that occur in every-day life. Comte affirmed that the only way to really understand the human mind, was to begin with man in the mass. Mr. Benzon, known for some time in racing and other charming circles by the euphonious designation of the "Jubilee Plunger," has just been tried at Nice for the common, vulgar, and criminal offences of paying with cheques when he had no effects at his bank, and of forging another man's name to a cheque for a thousand pounds. So far as Benzon himself is concerned, we should no more have thought of calling attention to his forgery, than to the conduct of a butcher's clerk who had forged his master's name for half-a-crown. The behaviour of any brainless youth of twenty-three can ordinarily be of little importance to the public, and that importance is certainly not increased by the silly creature's possession of two or three hundred thousand pounds. What is of importance is the conduct of a whole body of persons at Benzon's trial. Among those were the Marquis of Aylesbury, Captain Day, Mr. White, Mr. John Howitt, and the British Consul at Mentone. The peculiar defence those persons thought proper to make on behalf of a self-confessed forger, demands a little consideration. They seem to have thought that because Benzon was rich and a fool, therefore his crimes were to be considered as mere follies and overlooked as other follies might be. When Benzon forged he was "in despair." Exactly! But which of those men would have made such special pleading on behalf of a City clerk who should have forged his master's name for fifty pounds to save his wife and family from being turned out of house and home? The point that touches a mental physiologist is this: that all those defenders of Benzon were English to begin with, and therefore, presumably, men of some common sense and character. They were, moreover, men of education, and had at least been instructed in that code which is supposed to be the catechism and bible of the "English gentleman." Possibly, too, some of them had occasionally received hints and glimmerings of a code known to moral science which is higher than even that of the English gentleman. Why then did those "English gentlemen" astonish and confound foreigners by displaying a knowledge of morals and jurisprudence of which an Aztec or a Central African dwarf might justly have been ashamed? It is clear that if the "gentlemen" of the country display such results of education as this, physiological science must insist upon establishing a new order of things at all our public schools.

**Influenza.**

In The Hospital for December 28th last it was observed: "People as a rule are better fed, better clothed, better housed, and live under better sanitary conditions than they did, even a few years back, and they are therefore better able to withstand the onslaught of influenza or any other epidemic disease. Avoiding chill is the great preventive means; warmth, diaphoretics, expectorants, and quinine are the principal curative measures; and avoiding chill after an attack is the chief thing to attend to in order to escape unpleasant ulterior consequences." Is it possible that we are mistaken? We ask this question because we have seen at least fifty things advertised as cures for influenza, and at least as many more advertised as preventives. As prevention is better than cure, let us take preventives first. A dealer in shell fish placards that the best things to strengthen the system when it has fallen below par, and so escape influenza, are oysters. Now oysters are not bad things for those who like them, and are supposed to be "strengthening." On the other hand there are many who cannot digest oysters. And if an oyster happens to develope a "ptomaine," why then the consumer thereof may be attacked by a worse malady than influenza (vide Hospital Retrospect, December 28). Respirators and chest protectors have also come to the front as preventives. Well, we have nothing to say against these contrivances, but unfortunately we know of several votaries of respirators and chest protectors not escaping the prevailing epidemic. Then we are advised to study our health during the present unhealthy season by taking somebody's paregoric, as a certain preventive means. Now we all know that paregoric is a very good medicine. It is an old remedy, and has stood its ground many years. But to believe it will alone prevent influenza, whether made up into mixtures, or pills, or powders, or even lozenges, demands a degree of faith equal to that required for moving mountains! Compounds of quinine and antipyrin have of course come in for laudation as anti-influenza medicines. We have not, however, seen these remedies offered at the refreshment table, as recently pictured by *Punch*. Something called "Alofas," which it appears is "herbal," is recommended as a certain preventive and as a certain cure. It is said not to contain either quinine, opium, or any other poison, and to be equally efficacious for consumption, bronchitis, pneumonia, asthma, whooping-cough, measles, and fevers. It is unfortunate quinine and opium are mentioned as poisons, otherwise Alofas-ists might have quoted Dryden in favour of their remedy and said, "He 'scapes best who, nature to repair, draws physic from the fields." A positive cure is also "Koosh bitters." We do not quite know what "Koosh bitters" are. Koosh in the Eastern dialect means happy, content, pleasant. Well, one requires to be made happy and jovial when suffering from influenza. But whether "Koosh bitters" has the power of raising the low spirits, often so developed in the influenza-stricken, we take leave to doubt. But a sure prophylactic is placarded to be the juice of oranges. Certainly orange juice will not do us any harm, even when a patient has got the influenza, and we rather lean to the oranges than the Koosh bitters. Now all the advertisers of these certain cures, and of many others also, are apparently philanthropists! They announce their remedies for the good of the human race. But we think the old lines are as applicable to them as to the legitimate practitioners. "The creature does it for his dirty fee, and not for any love of you or me!" We wish the public could be brought to believe that there is no such thing as a universal medicine. In fact there is no universal medicine for even influenza. For the benefit of those who pin their faith on quack medicine we paraphrase the following well-known lines:—

For men are brought to worse distresses
By patent medicines than diseases,
And therefore commonly recover
When taking patents they give over.

